# Left-handers look before they leap: handedness influences reactivity to novel Tower of Hanoi tasks

**DOI:** 10.3389/fpsyg.2015.00058

**Published:** 2015-02-03

**Authors:** Lynn Wright, Scott M. Hardie

**Affiliations:** Evolutionary and Biological Approaches to Behaviour Research Group, Division of Psychology, Abertay UniversityDundee, UK

**Keywords:** handedness, Tower of Hanoi, task complexity, novelty, state anxiety, trait anxiety

## Abstract

A sample of 203 task naïve left- and right-handed participants were asked to complete a combination of the 3- and 4-disk Towers of Hanoi (ToH), manipulating novelty and complexity. Self-reported state anxiety and latency to respond (initiation time) were recorded before each ToH. Novelty had a major effect on initiation time, particularly for left-handers. Left-handers had a longer latency to start and this was significantly longer on the first trial. Irrespective of hand-preference, initiation time reduced on the second trial, however, this was greatest for left-handers. Condition of task did not systematically influence initiation time for right handers, but did for left-handers. State anxiety was influenced by task novelty and complexity in a more complicated way. During the first trial, there was a significant handedness × number of disks interaction with left-handers having significantly higher state anxiety levels before the 3-disk ToH. This suggests that the initial reaction to this task for left-handers was not simply due to perceived difficulty. On their second trial, participants completing a novel ToH had higher state anxiety scores than those completing a repeated version. Overall, left-handers had a larger reduction in their state anxiety across trials. Relating to this, the expected strong positive correlation between state and trait anxiety was absent for left-handed females in their first tower presentation, but appeared on their second. This was driven by low trait anxiety individuals showing a higher state anxiety response in the first (novel) trial, supporting the idea that left-handed females respond to novelty in a way that is not directly a consequence of their trait anxiety. A possible explanation may be stereotype threat influencing the behavior of left-handed females.

## INTRODUCTION

Research conducted from the 1970’s through to the early 1990’s examining the relationship between handedness and anxiety has produced a number of inconsistent and inconclusive findings. A common finding is that left-handers are more anxious, and worry more, than their right-handed counterparts (e.g., Orme, 1970; Hicks and Pellegrini, 1978; Davidson and Schaffer, 1983; Dillon, 1989). More specifically Orme (1970) found that left-handers reported themselves to be more introvert and shy than right-handers, Hicks and Pellegrini (1978) reported that left- and mixed-handers were significantly more anxious and Davidson and Schaffer (1983) reported higher trait anxiety levels in left-handers. Additional research around this time focussed upon the relationship between consistency of handedness (consistent versus inconsistent handedness) and anxiety. Wienrich et al. (1982) reported that consistent handers (irrespective of a left or right preference) had higher levels of anxiety than inconsistent handers, and Merckelbach et al. (1989) reported that consistent right-handers demonstrated higher social anxiety than left-handers. On the other hand, Mueller et al. (1991) examined differences in test anxiety between left- and right-handers and found that high-test anxiety did not affect left-handers any more than it affected right-handers. Other research has found no relationship between handedness and anxiety (e.g., French and Richards, 1990; Beaton and Moseley, 1991).

However, there has been a recent resurgence in research examining the relationship between handedness and anxiety. These studies have attempted to address some questions left unanswered by previous research. One key aspect of this recent research has been the use of the State Trait Anxiety Inventory (STAI; Spielberger et al., 1983) as the chosen measure. The STAI is arguably a good measure as it has a solid history of use in both clinical (e.g., Karch et al., 2008) and general psychological research (e.g., Baeken et al., 2011), and has good reliability (Bieling et al., 1998; Vautier and Pohl, 2009). The STAI has been designed to capture two main aspects of anxiety. State anxiety is a response to a given situation, and exists as an emotional response elicited by the situation, and expressed as a transient state of subjective worry, apprehension and general nervousness (Gerstorf et al., 2009; Roup and Chiasson, 2010). Tovilović et al. (2009) describe Trait Anxiety as a stable individual tendency to respond anxiously to all situations, and argue that this is really a measure of the likelihood that the individual will express state anxiety in a given situation. Although these two measures are conceptually linked, they are only moderately positively correlated (average correlation of 0.65, according to Spielberger et al., 1983). State anxiety is arguably the most appropriate measure to focus on, as it is more closely related to immediate behavioral responsiveness (Tovilović et al., 2009). Wright and Hardie (2012) examined state and trait anxiety differences between left- and right-handers. In contrast to previous studies (e.g., French and Richards, 1990) state anxiety was measured within the context of an experimental situation (i.e., introducing a mildly stressful scenario, allowing participants to have something to react to). Wright and Hardie (2012) found that left-handers reported higher levels of state anxiety but there was no difference in trait anxiety. They also demonstrated that when Trait Anxiety was controlled for, left-handers still showed a higher level of state anxiety compared to right-handers. This supports the notion that state anxiety differences are the most appropriate measurement to record when examining the reaction to a particular situation. Lyle et al. (2013) investigated the relationship between state anxiety, trait anxiety, worry and consistency of handedness. They found that inconsistent right-handers had lower levels of state and trait anxiety than consistent right-handers. In left-handers there was no relationship between consistency of handedness and anxiety, but inconsistent left-handers had higher levels of anxiety than inconsistent right-handers. While the rationale behind this difference remains unclear, Lyle et al. (2013) suggest that left-handers and right-handers may differ in terms of what triggers anxiety, and that this could “differ based on subjective differences in environmental experiences” (p. 14).

Studies carried out with non-human primates and human infants offer additional support to the relationship between handedness and anxiety. Westergaard et al. (2000) reported that high cortisol levels at 6 months in rhesus macaques were predictive of a left-hand bias at both 6 and 12 months of age. Adding to this, Westergaard et al. (2001) found an association between a left-hand preference and higher levels of the stress hormone cortisol in infant rhesus monkeys. Based on these findings Westergaard et al. (2001) argue that greater stress during infancy can *cause* a left-handed preference in rhesus monkeys. However, an alternative explanation of this finding might be that left-handedness, and thus right hemisphere motor dominance, increases anxiety and stress rather than stress causing the hand preference.

Very little research exists examining the relationship between hand preference and novelty. Of these studies, many involve non-human participants, and several report that if a task is new or unnatural to a left-hander this will increase their anxiety levels. For example, Cameron and Rogers (1999) found that there was a difference between left- and right-handed marmosets in their response behavior toward a novel object. They found that left-handers took significantly longer to approach and touch the novel object. Rogers (1999) replicated this finding and reported a difference in approach behavior to a novel object between left-handed and right-handed marmosets. Gordon and Rogers (2010) also found that right-handed marmosets were quicker to approach and interact with novel stimuli while Braccini and Caine (2009) found that left-handed marmosets took longer to approach and interact with novel food. These findings extend earlier work by Hopkins and Bennett (1994) who reported that left-handed chimpanzees were slower to approach novel objects than right-handed chimpanzees. However, Watson and Ward (1996) investigated temperament and problem-solving in the small-eared bush baby and found that left-handed bush babies were *less* inhibited in their approach to novel objects than right-handed subjects.

Thus, it appears that the introduction of a novel object may differentially influence the approach behavior of left-handers and right-handers. Rogers (1999) suggested that these findings could be explained by the differences in hemispheric specialization for processing novel stimuli and controlling emotional responses. She proposed that the left hemisphere controls exploratory behavior while the right hemisphere is associated with inhibitory or avoidance behavior. This would suggest that right-handers would be influenced by the dominant left hemisphere and would be more likely to demonstrate exploratory behavior, while left-handers would be more likely to be controlled by the right hemisphere and demonstrate inhibitory behavior. Supporting this notion is work by Davidson and colleagues (e.g., Davidson, 1985, 1992, 1995, 1998) linking behavioral avoidance and behavioral inhibition to the right-hemisphere. Sutton and Davidson (1997) also argue that the left-hemisphere is implicated in approach behavior. This suggests a model of hemispheric specialization in terms of interacting with the world that links the right-hemisphere to avoidance and the left-hemisphere to approach (see Rutherford and Lindell, 2011 for a review). In terms of evidence for the right-hemisphere, Shackman et al. (2009) found that individuals that are high in self-reported behavioral inhibition show an increased right dorsolateral prefrontal cortex resting activity, compared to low inhibited individuals. This lateralised pattern is further supported by studies linking the right-hemisphere to infants’ temperamental shyness, anxiety, and behavioral inhibition (Schmidt et al., 1999; Fox et al., 2001). Assuming that measures of lateral preference are also indicators of hemispheric dominance (Kinsbourne, 1997; Jackson, 2008) then a lateral preference is indicative of a preference for the contralateral hemisphere. For example, left-handers have been shown to self-report themselves as more behaviorally inhibited than right-handers (Wright et al., 2009). Arguably, this relationship between left-handedness and behavioral inhibition has become relatively well established, covering comparative evidence (Cameron and Rogers, 1999; Rogers, 2009), studies using self-reports (Hardie and Wright, 2013, 2014; Lyle et al., 2013) and through a series of behavioral studies (Wright et al., 2004, 2013; Wright and Hardie, 2011).

Although this work supports the proposal that the right hemisphere is associated with fear and avoidance behavior and is linked to the inhibitory system, Goldberg et al. (1994) have suggested an alternative explanation. They argue that the right hemisphere is specialized for novelty and that this hemisphere is more spontaneous, unreflective and does not effectively organize information but instead uses a type of trial and error system. Goldberg et al. (1994) suggest that the left hemisphere is concerned with a preference for familiarity and is more reflective and organized when processing information. Goldberg’s (2001) work does not systematically evaluate possible hemispheric differences related to hand preference. However, Goldberg (2001) himself suggests that left-handers appear to be more responsive to novelty than right-handers, and have a more varied distribution of cognitive processing, including a reversal in this set-up. Although there is little actual evidence to support this, it is possible that such differences in processes may contribute to differences when confronted with novelty. Work by Piper et al. (2011) provides evidence that left-handers may process novel situations differently from right-handers. During the novel image detection phase of the *‘novel-image novel-location’* spatial learning paradigm, left-handers were significantly more sensitive to changes, correctly noticing more insertions and details relating to the change.

Another factor that has to be taken in to account when considering anxiety levels and problem-solving is task complexity. A straightforward interpretation would argue that it is more likely that higher levels of anxiety will be produced when a task is more complex (Hembree, 1988). Contrary to this, Druckman and Swets (1988) stated that simple tasks actually require a high state of arousal by participants in order for them to remain focussed on the task. They add that as task complexity increases the level of arousal should decrease. Fink and Neubauer (2004) suggest a relationship between task complexity and stress in introvert/extravert participants. They reported that the easier a task was, the more likely introverted participants were to display lower cortical activation (suggesting that they were not as stressed). However, in more complex test conditions introverts showed higher cortical activation than extraverts.

In order to understand relationships between handedness and approaches to problem-solving we have investigated behavioral differences between left- and right-handers in novel tasks (Wright et al., 2004, 2013; Wright and Hardie, 2011). For example, Wright et al. (2004) found that left-handers took significantly longer to begin the 3-disk Tower of Hanoi (ToH) task. We proposed that one explanation for differences in approach behavior between left- and right-handers could be that left-handers might be experiencing higher levels of state anxiety in novel situations (Wright and Hardie, 2012).

The ToH, is cited as a commonly used test of executive function (e.g., Anderson and Douglass, 2001; Lezak et al., 2004), and executive functioning itself is a term used to describe a collective set of higher order cognitive functions (Beratis et al., 2013). These are thought to include inhibition, planning, working memory and cognitive flexibility (Goldstein et al., 2014). Work examining performance on the ToH, has strongly linked it to inhibition (Miyake et al., 2000), as overall success depends upon inhibiting moves that may appear to be correct but are wrong. Many studies use the ToH in the context of learning and memory, but few studies have systematically compared the 3- and 4-disk versions of the task. Mataix-Cols (2003) used a single presentation of the 3-disk version, but multiple presentations of the 4-disk task, and found that subclinical obsessive-compulsive (OC) individuals were poorer at solving the 3-disk version, although few differences were found in the 4-disk performance. Many studies make use of repeated trials of the ToH, and rarely report first move data when used in a novel situation. Contrary to this, Bustini et al. (1999) reported mean ‘planning time’ (i.e., time to make first move) for multiple trials of both 3- and 4-disk versions, comparing schizophrenic patients to matched controls. In this study, the patients had a longer mean planning time in both versions, but it was not significantly longer and only controls had a shorter time for the 3-disk version. Importantly, only right-handers were tested in this study. Guevara et al. (2013) examined developmental effects, again with only right-handed participants aged between 11 and 30 years old. They modified the 3-disk ToH with an additional rule – participants can move the disk to an adjacent peg only (i.e., no peg can be skipped over), increasing the solution to 26 moves. It was shown that time to make the first move averaged around 2–3 s, with no between group differences. Therefore the relationship between tower version and ‘time to make the first move’ is not straightforward. In the current study we will investigate these concepts further by manipulating both the novelty and complexity levels of the Tower of Hanoi Task, while adding the additional factor of handedness. Each participant will be asked to complete the Tower of Hanoi twice and will be in one of four conditions.

### CONDITION 1

Participants complete the 3-disk task, followed by a second 3-disk task (3–3) – novel verses non-novel version of the task and the simplest version of the task so complexity does not change.

### CONDITION 2

Participants complete the 3-disk task, followed by the 4-disk task (3–4) – simple and novel version of the task is completed first then the complexity increases in the second task and the task is slightly different due to the number of disks changing but the rules are the same.

### CONDITION 3

Participants complete the 4-disk task, followed by a second 4-disk task (4–4) – novel verses non-novel version of the task but a more complex version of the task (so again complexity does not change).

### CONDITION 4

Participants complete the 4-disk task, followed by the 3 disk task (4–3) – more complex but novel version of the task is completed first then the complexity decreases in the second task and the task is slightly different due to the number of disks changing but the rules are the same.

State anxiety and trait anxiety levels will be measured along with degree and direction of hand preference. On each Tower of Hanoi task time taken to move the first disk, number of moves taken and task completion time will be recorded. It is hypothesized that

### HYPOTHESES

#### Novelty

1 State anxiety levels will be higher and initiation time will be longer in left-handers when they complete the Tower of Hanoi for the first time only.

#### Complexity

2a. State anxiety levels will be higher and initiation time will be longer in left-handers when they complete the more complex 4-disk Tower of Hanoi.
2b. If complexity is important, when this increases on the second trial (i.e., 3–4) the state anxiety levels and initiation times should increase, compared to when this decreases on the second trial (i.e., 4–3).

## MATERIALS AND METHOD

### PARTICIPANTS

Two hundred and three participants took part in the study, all were university staff and students. Eighty-six participants were left-handed (39 males and 47 females) and 117 were right-handed (54 males and 63 females). The modal age category was 18–29 years. The Tower of Hanoi (ToH) task was completed twice by each participant where levels of novelty and complexity were manipulated (3–3; 3–4; 4–4; 4–3 disks). Participants were randomly assigned within their sex and handedness groups into conditions. All participants gave their informed consent to participate in the study. The study was approved by the School of Social and Health Sciences Ethics Committee and abided by the ethical regulations of the British Psychological Society.

### MATERIALS AND APPARATUS

#### State Trait Anxiety Inventory (Spielberger et al., 1983)

The state anxiety questionnaire consisted of 20 short statements. The directions on the questionnaire required participants to answer according to how they felt *right at that moment*. State anxiety statements included ‘I am tense’ and ‘I feel calm’ and these were answered on a four-point Likert scale ranging from ‘1 = not at all’ to ‘4 = very much so.’ Ten of the 20 statements were reverse scored and total scores ranged between 20 and 80. The trait anxiety scale consisted of another 20 statements which were also answered on a four-point Likert scale ranging from ‘1 = almost never’ to ‘4 = almost always.’ Directions instructed participants to read each statement and answer in relation to how they *generally feel*. Statements this time included ‘I lack self-confidence’ and ‘I am calm, cool and collected.’ Responses were totalled and scores ranged from 20 to 80. A score of 20 on both scales indicated low anxiety levels and 80 indicated high anxiety levels.

#### Tower of Hanoi

The Tower of Hanoi Task consisted of three pegs and up to four colored disks stacked on one of the pegs. Counterbalancing was carried out so that half of all left-handed and right-handed participants began the task with the disks stacked on the left peg and worked to move all of the disks to the last empty peg on the right. The other half of the left- and right-handers began with the disks stacked on the peg on the right side and aimed to stack them all on the empty peg on the left. The disks were stacked on the peg in order of size with the largest one on the bottom and the smallest one on the top. The two empty pegs were used to move the rings from the full peg to the last empty peg. A cardboard cover was used to conceal the Tower of Hanoi to ensure that participants could not see it. A stopwatch with a split-time function was used which allowed the initial first move time to be stored alongside the total completion time. To ensure consistency the same researcher measured the initiation and completion times on the Tower of Hanoi. The process of measuring and recording the ToH variables was identical to Wright et al. (2004). Written instructions were given to participants outlining the rules of the task and depicting the initial state and goal state. Participants were instructed that they were going to see three pegs and on one of the pegs there would be a number of disks (either three or four depending upon the condition) stacked on it (there were separate instructions for the 3-disk and the 4-disk trials). The rules were that only 1-disk could be moved at a time, a larger disk could not be placed on a smaller disk and the participant should only use their dominant hand to carry out the task. A different set of instructions were given to participants when they did the Tower of Hanoi for the second time. The instructions again outlined the rules of the task and showed the initial state and goal state but either stated that the participant was going to be asked to do exactly the same task again (if they were in the 3-disk, followed by 3-disk; or 4-disk, followed by 4-disk condition) or that they would be asked to do a ***similar*** task but that a disk would be added (if they were in the 3-disk, followed by 4-disk condition) or taken away (if they were in the 4-disk, followed by 3-disk condition). The optimal solution for the 3-disk ToH was seven moves and for the 4-disk ToH, 15 moves.

#### Handedness questionnaire

Following Peters’s (1998), Wright et al. (2004) handedness questionnaire was used to measure participant’s handedness. The original version is a 25-item scale scored using a five point Likert scale (left-hand always, left-hand mostly, either hand, right-hand mostly and right-hand always). The five points on the scale are assigned values from -2 (always use the left hand) through to 2 (always use the right hand) and each item is scored individually then totalled to give an overall handedness score. A total positive value indicates a right-hand preference and a total negative value indicates a left-hand preference.

### PROCEDURE

Participants were asked to complete Peters (1998) handedness questionnaire. Participants were then given a copy of the instructions for the novel problem-solving task (either the 3-disk or 4-disk Tower of Hanoi). After reading the Tower of Hanoi instructions participants were asked to complete the state anxiety questionnaire of the STAI to measure current levels of anxiety. They were instructed to answer the questions according to how they felt *right at that time*. Once this was completed participants were instructed to solve the Tower of Hanoi (3- or 4-disk depending on the condition that they were assigned to) with their preferred hand. The Tower of Hanoi was concealed with a large cardboard cover and this was removed when the participant was ready to begin the task. When the participant made *physical contact* with the first disk the experimenter recorded the initiation time on the split-time stopwatch. The experimenter also kept a note of the number of moves the participant took to solve the Tower of Hanoi. When the participant had successfully solved the Tower of Hanoi the stopwatch was stopped and the total time taken to complete the task was recorded. In order to create a delay between the two tasks and purely to act as a distractor, participants had their digit ratio measured on both hands. This created a gap of ∼5 min. A second set of Tower of Hanoi instructions was then given to participants. These instructions differed depending on the condition that each participant was assigned to. Participants who were in the condition where they did the 3-disk or 4-disk Tower of Hanoi twice (3–3; 4–4) were given an identical set of instructions to the ones they received the first time except this time it was emphasized that the task was exactly the same as they did the first time and that the rules were the same as the first trial. Those who completed the 3-disk Tower of Hanoi or 4-disk Tower of Hanoi first were given a set of 4-disk and 3-disk Tower of Hanoi instructions respectively (3–4; 4–3) which again outlined the rules and showed a picture of the initial and goal states. When participants had read the instructions they were asked to fill in a second state anxiety questionnaire and then complete the second trial of the Tower of Hanoi. The side of the initial disk stack was counterbalanced across all participants so, for example, half of the left-handed males started from the right when doing the 3-disk Tower of Hanoi and the other half started on the left hand side. This was the same for the other sex and handedness groups across the 3-and 4-disk trials. Again initiation time, number of moves and completion time were recorded. Only participants who had never solved the Tower of Hanoi before were included in the sample to ensure that the task remained novel to all participants throughout the experiment. Finally, the trait anxiety questionnaire was completed and participants were fully debriefed.

## RESULTS

**Table 1** summarizes the results for both tower presentations, listed separately for left- and right-handers. For the first tower, all participants were naïve and so the tower was novel, but there were two conditions differing in complexity (3-disk versus 4-disk). For the second tower, there was the added complication of whether the task was the same (3–3, 4–4), made easier (4–3) or more difficult (3–4).

**Table 1:** Summary of results.

			Trait anxiety	State anxiety	Initiation time	Moves	Time
**Tower 1**
	3-Disk	**Total**	**40.1 (9.0)**	**36.7 (8.7)**	**4.4 (4.8)**	**11.9 (6.2)**	**50.6 (37.4)**
		Left	41.8 (8.4)	39.4 (8.3)	5.8 (5.9)	11.4 (86.9)	45.6 (33.2)
		Right	38.9 (9.3)	34.9 (8.5)	3.4 (3.6)	12.2 (5.7)	54 (40)
	4-Disk	**Total**	**40.9 (10.6)**	**34.8 (10)**	**4.0 (3.9)**	**26.9 (10.2)**	**116.6 (73.2)**
		Left	39.5 (9.4)	34.2 (9.7)	4.5 (4.4)	26.7 (9.8)	110.5 (65.1)
		Right	42 (11.4)	35.3 (10.3)	3.6 (3.4)	27.1 (10.7)	121.4 (79.3)
**Tower 2**
	3–3	**Total**	**39.8 (9.6)**	**29.3 (7.6)**	**1.3 (.5)**	**10.1 (4.4)**	**33.5 (20.6)**
		Left	41.8 (8.6)	30.6 (7.1)	1.5 (.7)	9.3 (2.3)	31.7 (19.5)
		Right	39.0 (10)	28.8 (7.8)	1.2 (.4)	10.5 (5)	34.2 (21.2)
	3–4	**Total**	**39.7 (8.8)**	**34.3 (7.5)**	**2.5 (2.4)**	**27.4 (13.4)**	**100.7 (93.4)**
		Left	41.9 (8.4)	35.4 (5.9)	3.0 (2.9)	25.3 (12.1)	93.6 (82.5)
		Right	37.4 (8.7)	33 (8.9)	1.9 (1.6)	29.6 (14.5)	108.3 (105)
	4–4	**Total**	**39.0 (9.1)**	**30.5 (8.5)**	**2.1 (1.2)**	**25.5(11.6)**	**63.5 (44.2)**
		Left	37.6 (8.2)	29.7 (7.9)	2.2 (1.4)	25.6 (11.6)	62 (45.3)
		Right	40.4 (10)	31.4 (9.2)	2.0 (1.1)	25.3 (11.8)	65.2 (43.8)
	4–3	**Total**	**44.0 (11.4)**	**34.7 (10.9)**	**1.4 (0.9)**	**12.3 (9.3)**	**39.1 (30.2)**
		Left	42.9 (10.8)	34.1 (12.8)	1.7 (1.4)	11.3 (3.7)	34.8 (18.8)
		Right	44.5 (11.8)	35.1 (10)	1.2 (0.5)	12.8 (11.3)	41.4 (34.9)

### STATE ANXIETY

#### State anxiety on first tower (novel task)

We initially examined the difference between left- and right-hander’s state anxiety levels (state anxiety before completing the ToH for the first time) irrespective of the number of disks the participant completed in the trial, in order to look for a *general* effect of the task on anxiety. There was no significant difference between state anxiety scores of left- (*m* = 36.7) and right-handers (*m* = 35.1) before their first Tower of Hanoi task *t*(201) = 1.29, *p* = 0.199. **Table 1** indicates that left-handers had higher state anxiety levels than right-handers when completing the three-disk ToH in the first task but right-handers have slightly higher state anxiety scores before completing the four disk ToH in the first task.

**FIGURE 1:**
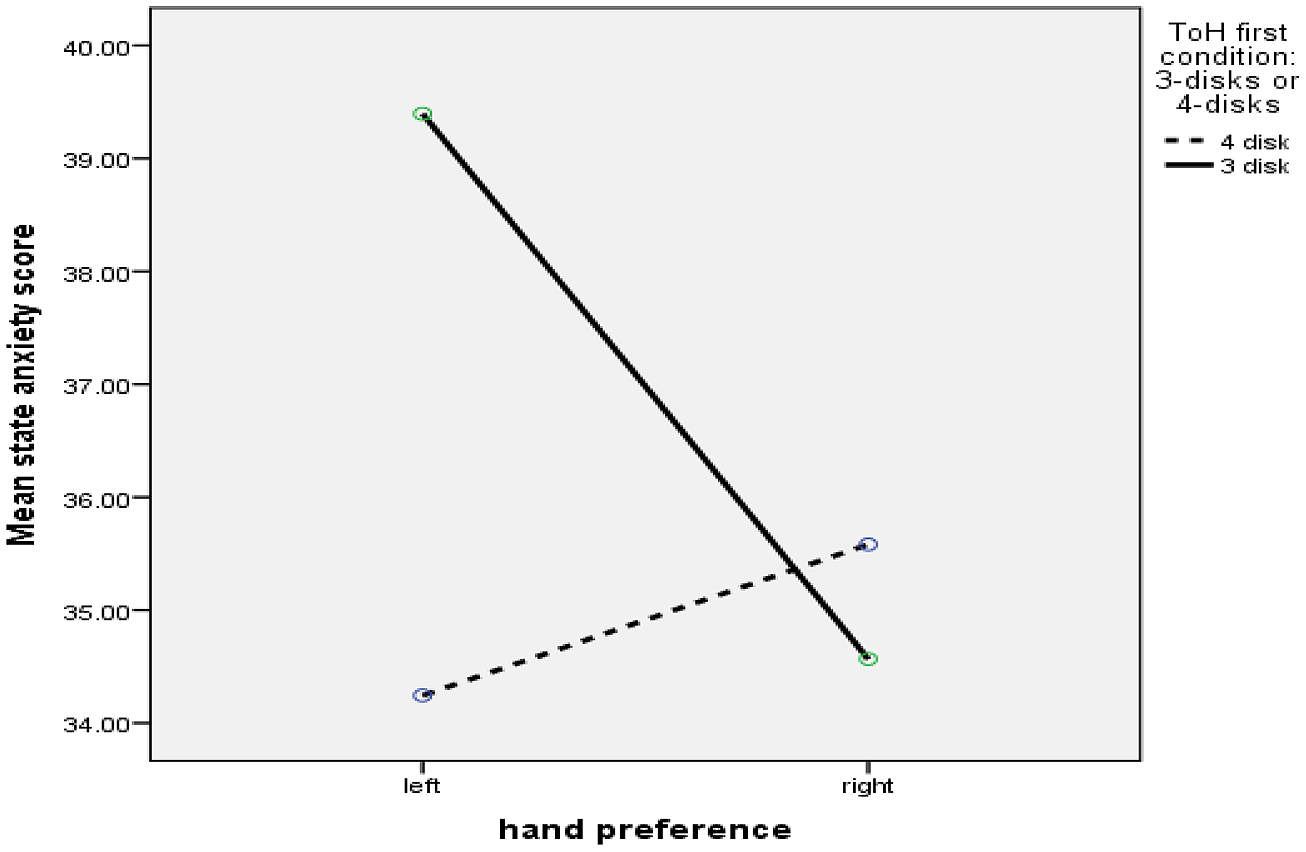
**Hand preference by disk number interaction (state anxiety scores)**.

A 2 (gender) × 2 (hand preference) × 2 (number of disks) between subjects ANOVA was carried out to investigate individual state anxiety scores before their first ToH task. There was no significant main effect of gender *F*(1,195) = 1.8, *p* = 0.18, hand preference *F*(1,195) = 1.46, *p* = 0.23, or number of disks *F*(1,195) = 3.27, *p* = 0.07. However, there was a significant interaction between handedness and number of disks *F*(1,195) = 5.0, *p* = 0.026, partial η^2^ = 0.03, observed power = 0.6. **Figure 1** shows that left-handers were most anxious prior to starting the 3-disk Tower of Hanoi. *Post hoc* pairwise comparisons (Tukey) showed that the only significant difference was between the left-handers (*p* = 0.05) where participants had a higher state-anxiety score when preparing to start the 3-disk tower.

#### State anxiety on second tower (novelty and complexity)

In order to investigate the hypotheses, response to the second tower can be examined in two main ways; the response to the second tower can be examined itself, followed by a comparison between the first and second trials. A 2 (hand preference) × 2 (number of disks) × 2 (Novelty) between subjects ANOVA was initially carried out to investigate state anxiety scores before the second ToH task. There was no significant main effect of hand preference *F*(1,195) = 0.09, *p* = 0.76, or number of disks *F*(1,195) = 0.23, *p* = 0.64. There was a significant main effect of novelty *F*(1,195) = 11.48, *p* = 0.001, partial η^2^ = 0.06, observed power = 0.9, where individuals encountering novel versions of the tower had significantly more state anxiety. There were no significant interactions.

### STATE ANXIETY DIFFERENCES (INFLUENCE OF CONDITION)

A 2 (hand preference) × 4 (condition) between subjects ANOVA was then used to investigate the mean difference in state anxiety between the first and second trials. There was a significant main effect of hand preference *F*(1,195) = 4.9, *p* = 0.028, partial η^2^ = 0.03, observed power = 0.6, with left-handers showing a significantly larger drop in their state anxiety when encountering the task for the second time. There was a significant main effect of disk-condition *F*(3,195) = 7.6, *p* < 0.001, partial η^2^ = 0.1, observed power = 1. Tukey *post hoc* tests were carried out to further investigate the significant state anxiety difference scores between the four disk-conditions. **Table 2** indicates that the largest reduction was for the 3–3 condition (easy task completed twice), there were significant state anxiety score differences between the 3–3 and 4–4 (*p* < 0.001) and between the 3–3 and 3–4 (*p* = 0.006) conditions. No other comparisons were significant.

**Table 2:** Mean state anxiety and initiation time difference scores (Time 1 – Time 2) between the first and second Towers of Hanoi (novel vs. not novel) when disk number is considered.

Hand-preference	Condition	Mean state difference*	Total	Mean initiation difference*	Total
Left	3–3	8.6 (8.9)		6.2 (8.1)	
Right	3–3	6.0 (5.7)		2.3 (3.7)	
	**3–3 Total**		**6.8 (6.8)**		**3.4 (5.6)**
Left	4–3	6.3 (10.2)		1.9 (3.3)	
Right	4–3	2.7 (7.3)		1.4 (3.6)	
	**4–3 Total**		**4.0 (8.5)**		**1.6 (3.4)**
Left	4–4	1.0 (5.6)		1.6 (1.9)	
Right	4–4	1.9 (3.5)		2.5 (3.6)	
	**4–4 Total**		**1.4 (4.7)**		**2.0 (2.9)**
Left	3–4	4.0 (5.4)		3.9 (6.1)	
Right	3–4	1.2 (4.2)		1.5 (2.7)	
	**3–4 Total**		**2.7 (5.0)**		**2.4 (4.4)**

*A state anxiety difference score was calculated by subtracting state anxiety 2 from state anxiety 1 scores. A higher score indicates a larger reduction, between ToH1 and ToH2.

### INITIATION TIME

#### Initiation time on first tower (novel task)

There was a significant difference between initiation times scores of left- (*m* = 5.1 s) and right-handers (*m* = 3.5 s) before their first Tower of Hanoi task *t*(201) = 2.7, *p* = 0.007, with left-handers taking significantly longer. **Table 1** includes figures broken down by disk number. Left-handers took longer to move the first disk in general and the longest initiation time was taken by left-handers in the 3-disk condition.

A 2 (gender) × 2 (hand preference) × 2 (number of disks) between subjects ANOVA was carried out to investigate the time taken to move the first disk of the first ToH task (initiation time). There was a significant main effect of gender, *F*(1,195) = 11.4, *p* = 0.001, partial η^2^ = 0.1, observed power = 0.9 There was a significant main effect of hand preference *F*(1,195) = 6.7, *p* = 0.010, partial η^2^ = 0.03, observed power = 0.7 (with left-handers taking significantly longer to move the first disk). There was no significant main effect of number of disks *F*(1,195) = 0.81, *p* = 0.37. There was a significant interaction between gender and handedness *F*(1,195) = 3.9, *p* = 0.05. *Post hoc* analyses (Tukey) showed that female left-handers took longer to start than male left-handers (*p* = 0.003), and also both male (*p* < 0.001) and female (*p* = 0.004) right-handers. There were no other significant interactions.

#### Initiation time on second tower (novelty and complexity)

For initiation time during the second tower, there was once again a significant main effect of hand preference *F*(1,195) = 6.8, *p* = 0.01, partial η^2^ = 0.03, observed power = 0.7 (with left-handers taking significantly longer to move the first disk). There was also a significant main effect of number of disks *F*(1,195) = 16.4, *p* < 0.001, partial η^2^ = 0.1, observed power = 1, with a longer mean initiation time for the 4-disk task. There was no main effect of novelty *F*(1,195) = 1.1, *p* = 0.29. There were no significant interactions.

### INITIATION TIME DIFFERENCES (INFLUENCE OF CONDITION)

A 2 (hand preference) × 4 (condition) between subjects ANOVA was then used to investigate the mean difference in initiation time between the first and second ToH trials. There was a significant main effect of hand preference *F*(1,195) = 6.3, *p* = 0.013, partial η^2^ = 0.03, observed power = 0.7 (with left-handers having a significantly larger reduction in initiation time between trials). There was a significant main effect of condition *F*(3,195) = 3.6 *p* = 0.014, partial η^2^ = 0.05, observed power = 0.8, but individual pairings were not significantly different from each other. There was also a significant interaction between hand preference and condition, *F*(3,195) = 3.2, *p* = 0.026, partial η^2^ = 0.05, observed power = 0.7. Follow-up testing demonstrated that for right-handers there was no influence of condition *F*(3,113) ≤ 1, but for left-handers there was a significant effect of condition *F*(3,82) = 3.7, *p* = 0.015, partial η^2^ = 0.1, observed power = 0.8. As the largest initiation time differences was for the 3–3 condition (easiest task completed twice), Tukey *post hoc* tests indicated that there were significant initiation time score differences between the 3–3 and 4–4 (*p* = 0.019) and between the 3–3 and 3–4 (*p* = 0.031) conditions. No other comparisons were significant.

### TRAIT ANXIETY

The mean trait anxiety score for left-handers was 40.6 and was 40.4 for right-handers. A 2 (gender) × 2 (handedness) ANOVA revealed no significant main effects or interactions.

### PERFORMANCE

Performance on the Tower of Hanoi was examined using a 2 (gender) × 2 (handedness) ANOVA. There were no main significant effects of handedness or gender on number of moves or completion time for either first or second trial, but there was a handedness × gender interaction on the number of moves during the first trial [*F*(1,199) = 5.0, *p* = 0.026, partial η^2^ = 0.03, observed power = 0.6). However *post hoc* tests failed to reveal any differences between the combinations.

### RELATIONSHIP BETWEEN HAND PREFERENCES STRENGTH, ANXIETY, INITIATION TIME AND PERFORMANCE

As Lyle et al. (2013) found that strength of handedness was related to degree of anxiety (this relationship was only found for right-handers); it was decided to explore the inter-relationship between variables. The analysis presented here is focussed on the first tower, as this is where novelty, complexity and anxiety were easiest to compare. However, the same analysis was also done for the second tower and this is shown in **Table 5**.

**Table 3** outlines the relationship between the variables strength of hand preference, initiation time, number of moves and both state and trait anxiety. This was also carried out separately for hand-preference category and gender. For brevity, analysis will focus on correlations of 0.2 or above, as well as those common and/or divergent across the data set. It is noted that although these are significant, most of these are fairly weak correlations. As would be expected, there was a significant positive correlation between number of moves and time to solve, as well as a positive correlation between initiation time and time to solve. For left-handers, there was a negative correlation between state anxiety and number of moves (*r*86 = -0.245, *p* = 0.023), suggesting that a higher level of anxiety led to a lower number of moves in solving the tower. Right-handers showed no such relationship. In terms of gender, number of moves and time to solve was also positively correlated, but male initiation time and time to solve were not. Females also demonstrated a significant negative correlation between handedness score and initiation time, showing that an increasing strength of left-handedness was related to a slower initiation time. Also, females had a positive relationship between trait anxiety and number of moves, with increasing anxiety scores related to a larger number of moves. Contrary to expectations based on Lyle et al. (2013), there was no relationship between strength of handedness and either state or trait anxiety. However, this could be due to the fact that strength of handedness in the current study was treated as a continuous variable while Lyle et al. (2013) treated this variable dichotomously in to inconsistent and consistent categories for both left- and right-handers.

**Table 3:** Correlations between main variables, for Tower of Hanoi task irrespective of disk number.

Tower 1 – All	Number of moves	Time to solve	Handedness score	State anxiety	Trait anxiety
Initiation time	-0.033	0.223^**^	-0.152^∗^	0.099	-0.043
Number of moves		0.748^**^	-0.051	-0.057	0.157^∗^
Time to solve			0.038	0.106	0.157^∗^
Handedness score				-0.060	-0.009
State anxiety					0.534^**^
**Left-handers (*N* = 86)**
Initiation time	-0.163	0.243^*^	0.108	0.189	-0.062
Number of moves		0.704^**^	-0.164	-0.245^*^	0.096
Time to solve			-0.019	0.000	0.138
Handedness score				0.015	-0.060
State anxiety					0.370^**^
**Right-handers (*N* = 117)**
Initiation time	0.106	0.249^**^	-0.010	-0.025	-0.036
Number of moves		0.783^**^	-0.096	0.082	0.198^∗^
Time to solve			-0.035	0.183^∗^	0.169
Handedness score				0.097	0.081
State anxiety					0.641^**^
**Males (*N* = 93)**
Initiation time	-0.012	0.138	-0.024	-0.007	0.091
Number of moves		0.759**	0.090	-0.136	0.061
Time to solve			0.162	-0.008	0.118
Handedness score				0.032	0.102
State anxiety					0.601^**^
**Females (*N* = 110)**
Initiation time	-0.044	0.244*	-0.218*	0.139	-0.138
Number of moves		0.752**	-0.161	0.018	0.238*
Time to solve			-0.050	0.190*	0.174
Handedness score				-0.137	-0.089
State anxiety					0.471**

*Correlation is significant at the 0.05 level (two-tailed). **Correlation is significant at the 0.01 level (two-tailed).

A similar set of relationships were found across the second tower, except that initiation time had a stronger relationship with hand preference score (-0.245) and this was largely driven by females (-0.327). Left-handers also showed a relationship between initiation time and state anxiety in the second trial (0.266).

Potentially one of the main relationships of note was the expected positive correlation between Trait and State Anxiety, which was significant across all data sets, for both towers. However, for left-handers in the first tower the relationship was a lot weaker and it was found to be significantly different from the right-handed score (*z* = -2.57, *p* = 0.01). Females also had a lower correlation, but this was not significant. In order to better understand this, it was decided to further investigate the state and trait correlation split by both handedness and gender.

**Table 4** demonstrates that all hand and gender combinations have a significant relationship between state and trait anxiety, except for left-handed females who show no such relationship. However, in the second tower, they now show a significant correlation (0.374). This suggests that on the first trial of the Tower of Hanoi they were reacting differently.

**Table 4:** Correlation between State and Trait Anxiety, split by gender and hand preference.

	ToH1						ToH2	
	All	*N*	3-Disk	*N*	4-Disk	*N*	All	*N*
Female left-handers	0.073	47	0.052	23	0.057	24	0.374**	47
Male left-handers	0.617**	39	0.603**	19	0.613**	20	0.657**	39
Female right-handers	0.689**	63	0.697**	36	0.709**	27	0.641**	63
Male right-handers	0.592**	54	0.427*	26	0.672**	28	0.655**	54

*Correlation is significant at the 0.05 level (two-tailed). **Correlation is significant at the 0.01 level (two-tailed).

**Table 5:** Correlation between main variables, for second task irrespective of disk number.

Tower 1 – all	Number of moves	Time to solve	Handedness score	State anxiety	Trait anxiety
Initiation time	0.204*	0.313**	-0.245**	0.094	**-**0.031
Number of moves		0.652**	**-**0.028	0.036	**-**0.041
Time to solve			0.020	0.126	**-**0.030
Handedness score				**-**0.007	**-**0.009
State anxiety					0.589**
**Left-handers (*N* = 86)**
Initiation time	0.188	0.418**	**-**0.071	0.266*	0.099
Number of moves		0.762**	0.050	0.010	0.071
Time to solve			0.108	0.183	0.136
Handedness score				0.047	**-**0.060
State anxiety					0.495**
**Right-handers (*N* = 117)**
Initiation time	0.240**	0.230*	**-**0.099	**-**0.139	-0.210*
Number of moves		0.584**	0.107	0.049	**-**0.105
Time to solve			0.124	0.089	**-**0.127
Handedness score				0.087	0.081
State anxiety					0.645**
**Males (*N* = 93)**
Initiation time	0.301**	0.402**	**-**0.103	0.104	0.154
Number of moves		0.527**	**-**0.126	0.011	**-**0.056
Time to solve			**-**0.008	0.098	**-**0.004
Handedness score				**-**0.008	**-**0.008
State anxiety					0.651**
**Females (*N* = 110)**
Initiation time	0.145	0.259**	-0.327**	0.078	**-**0.153
Number of moves		0.803**	0.040	0.045	**-**0.037
Time to solve			0.051	0.154	**-**0.065
Handedness score				**-**0.122	**-**0.089
State anxiety					0.540**

*Correlation is significant at the 0.05 level (two-tailed). **Correlation is significant at the 0.01 level (two-tailed).

A final analysis was conducted, dividing Trait anxiety in High and Low (by use of a median split), whereby those above the median (39) were included in the former category. Each sex by handedness category was compared individually using an independent *t*-test. Full details are shown in **Table 6**. In nearly all cases there was a significant difference between High and Low trait anxiety groups, where the High groups showed significantly higher mean state anxiety in both towers (*p* = 0.004 or lower). The exception was for female left-handers and on the first tower only. The mean for the High Trait groups’ state anxiety was 38.5 (SD = 9.8) but was 37.6 (SD = 7.8) the Low Trait group, this meant that they were not significantly different on state anxiety, *t*(45) = 0.360, *p* = 0.720. On the second trial, the High group was now significantly higher (*m* = 37) compared to the Low group (*m* = 31.3), *t*(45) = 2.12, *p* = 0.039.

**Table 6:** Influence of trait anxiety category (high versus low) on State anxiety levels for both towers.

	Female left handers	Male left handers	Female right handers	Male right handers
**Tower 1**				
High trait anxiety	38.5 (9.6)	38.9 (8.7)	39.7 (7.8)	39.1 (10.4)
Low trait anxiety	37.6 (7.8)	30.7 (9.4)	30.0 (7.2)	31.1 (7.8)
Independent-*t*	0.360	2.8	5.1	3.2
*p-value*	0.720	0.008	<0.001	0.002
**Tower 2**				
High trait anxiety	37.0 (9.4)	33.3 (6.4)	36.8 (8.8)	36.5 (10.0)
Low trait anxiety	31.3 (8.8)	27.1 (6.3)	25.9 (5.6)	27.8 (6.0)
Independent-*t*	2.1	3.1	5.7	3.9
*p-value*	0.039	0.004	<0.001	<0.001

## DISCUSSION

It was hypothesized that state anxiety would be higher in left-handers on the first Tower of Hanoi trial only. The first hypothesis was not supported, as there was no overall significant state anxiety difference between left- and right-handers on the first ToH trial. However, there was a significant interaction between handedness and the number of disks which was influenced by the higher state anxiety levels of left-handers when they did the 3-disk Tower of Hanoi. When left- and right-hander’s state anxiety levels were examined on the second ToH trial there were no significant differences between them. It was also hypothesized that initiation times would be longer for left-handers on the first ToH trial only. Left-handers took significantly longer to move the first disk on the task than right-handers, however, we also found that left-handers took significantly longer to move the first disk on the second trial therefore this hypothesis was not supported. Additionally, for all groups in the second trial the initiation times were significantly faster. When taking the complexity of the Tower of Hanoi in to consideration it was hypothesized that state anxiety levels would be higher in left-handers when completing a more complex task. This hypothesis was not supported as the highest state anxiety was found in left-handers during the 3-disk task. However, there was a significant influence of complexity on state anxiety when the four conditions were examined. The largest reduction in state anxiety occurred when participants completed the simplest task (3–3) for the second time. There was also a general handedness effect with left-hander’s showing a significant reduction in state anxiety levels on the second ToH.

We also hypothesized that initiation time would be longer for left-handers when completing a more complex version of the ToH. This hypothesis was not directly supported, as initiation time was not influenced by the number of disks. However, there was a significant interaction between gender and handedness. Female left-handers took longer than the other groups to begin the task. There was still a significant main effect of handedness on the initiation time on the second task, but not for gender. As before there was a main effect of handedness across all conditions.

Although task novelty strongly contributed to left-handers’ delay in initiation, it was still found (but to a lesser degree) on the second trial. This supports the view that left-handers are more behaviorally inhibited than right-handers (Wright et al., 2009) but also suggests that the nature of the task, in particular novelty and difficulty, may have an effect. The significant handedness × disk condition effect on changes in initiation time supports this view. For left-handers, the simplest task combination (3–3) had the largest drop in initiation time, and this was larger compared with when the second task complexity increased (3–4), and also when the combination was most difficult (4–4). This indicates that for left-handers, initiation time is sensitive to task complexity; when the task is not novel and simple, their initiation time is fastest.

Looking at behavioral inhibition differences, we have again shown that left-handers take longer to start a task, and this is most pronounced when the task is novel. This concurs with our previous finding on the 3-disk Tower of Hanoi (Wright et al., 2004) and a card-sorting task (Wright and Hardie, 2012) and follows expectations based on linking the right-hemisphere to inhibition and behavioral avoidance (Davidson, 1992, 1998; Sutton and Davidson, 1997). Similarly, in the context of a memory test, Lyle et al. (2012) found left-handers to be slower to respond but not less accurate. At least as far as novel situations are concerned, left-handers seem to pause longer than right-handers, but in the present case this did not have any direct influence on their performance on the Tower of Hanoi.

This supports the view that the longer initiation time is not taken up by planning the task, and that it is more likely to be a handedness related difference in assessing the situation (Piper et al., 2011). Further support may be gained from an examination of effects. Females tended to take longer to start, and female left-handers took longer than all other groups, and although it has been suggested that females are poorer at visuo-spatial tasks than males, it has also been proposed that gender does not significantly influence performance differences on the ToH (e.g., Salnaitis et al., 2011). Once again, the fact that females were not any worse in actual performance suggests that gender may be influencing performance style rather than ability to solve the task. For example, Hugdahl et al. (2006) used fMRI during a 3-D mental rotation task and found that males and females differ in terms of the processing strategies they use, with females using a verbal (language guided) approach contrasting with the perceptual (spatially guided) approach used by males. Although not tested in the present study, it remains possible that both left-handers and females may approach the solving of the task in a different way from males and right-handers.

On the other hand, the lack of a clear-cut anxiety difference was not expected as we had previously shown that left-handers exhibited a higher level of state anxiety (Wright and Hardie, 2012). Surprisingly, the first presentation of the simple (3-disk) tower elicited the highest level of state anxiety, which was shown by left-handers. The absence of a gender effect in anxiety is not surprising given the lack in previous studies where handedness was a major factor (e.g., Merckelbach et al., 1989; French and Richards, 1990; Wright and Hardie, 2012) but is in contrast to some other studies, including McLean and Anderson’s (2009) review, which showed females having a higher level of anxiety. The issue of anxiety and gender will be further considered when we examine the relationship between state and trait anxiety.

In common with the developers of STAI (Spielberger et al., 1983), numerous studies have found a positive correlation between state and trait anxiety (e.g., Abdel-Khalek, 1989; Carstensen et al., 2000; Vigneau and Cormier, 2008). Lyle et al. (2013) examined anxiety during a break from testing while carrying out a cognitive task (i.e., not immediately anxiety provoking) and found a strong positive correlation (0.80) across their balanced sample of left- and right-handers. However, they do not present data for handedness classes separately. In our previous research (Wright and Hardie, 2012) immediately after participants received the instructions for a computerized cognitive task (i.e., an anxiety provoking situation), we found that for both left- and right-handers, state and trait anxiety were significantly positively correlated (0.55 overall, with 0.57 for left-handers and 0.51 for right-handers). Gender and handedness were not separately presented in the original study, but re-analysis of the raw data has shown that female left-handers did not have a significant correlation between state and trait anxiety, while the other three groups did. This data was from a different sample from the present study, and suggests that in a novel and stress-inducing situation, left-handed females appear to show a state anxiety response that is not immediately related to their trait anxiety levels. Some support for females occasionally showing this kind of disconnection, comes from a study into maths anxiety, where females but not males had a mismatch between their state and trait anxiety levels (Goetz et al., 2013).

State anxiety has been shown to be a good measure of current anxiety, as it can chart differences in response before, during and after a stressor is presented (Harrigan et al., 1991). It also corresponds to experimental manipulations that either increase stress (e.g., lecturing to 200 people; Filaire et al., 2010) or decrease stress (e.g., using Yoga to relax; Subramanya and Telles, 2009). State anxiety itself may consist of two components, ‘worry’ and emotionality and the latter is considered to be equivalent to neuroticism (Mellanby and Zimdars, 2011). However, worry is thought to be the component that may influence performance (Hayes et al., 2008) and while in the current study there were no obvious performance effects, left-handers had the highest level of state anxiety on their first 3-disk task along with the longest initiation time. In all cases left-handers had the longest initiation time, but this was not always associated with a higher level of state anxiety. Thus, the relationship between handedness, anxiety and performance on the Tower of Hanoi is complicated, influenced by novelty, complexity and gender.

In contrast, trait anxiety may be a good predictor of general responsiveness (e.g., Bishop et al., 2004). For example, high trait anxiety has been linked with risk-avoidant decision-making (Broman-Fulks et al., 2014) but it may not be sensitive to changes in current stressors (e.g., Cesci et al., 2009). Trait anxiety is also thought to be a predictor of propensity to react in a vigilant and threat seeking manner (e.g., Mathews and McLeod, 2002) and as such, has been used a predictor of responsiveness. For example, trait anxiety differences (usually categorizing participants into a high versus low trait anxiety group) have been successfully used to predict between group differences in a number of tasks (Koster et al., 2005; Viaud-Delmon et al., 2011). Schlotz et al. (2006) showed that an association between cortisol and subjective performance pressure was mediated by trait anxiety – with no association at low levels but thereafter it was higher as trait anxiety increased. Trait anxiety also correlates with neuroticism (Luteijn and Bouman, 1988), so the degree that each measure is tapping into something different may be unclear, but theoretically the ‘worry’ aspect of state anxiety may best reflect the component of anxiety that is most strongly influenced by the current situation. In any case, as Wilt et al. (2011, p. 989) put it ‘Although trait anxiety may influence the level or probability of state anxiety, it is likely that trait and state forms of anxiety are not completely isomorphic; that is, trait and state anxiety may arise from different causes and have different consequences.’

Linking anxiety and behavior to the revised reinforcement sensitivity theory (rRST) may provide some additional clues. Wright and Hardie (2011) have proposed a link between handedness and degree of Behavioral Inhibition, in the context of Gray and McNaughton’s (2000) rRST. This theory describes personality in terms of three major interacting systems that influence action (Corr and McNaughton, 2008), and these are the behavioral activation system, or BAS, the fight-flight-freeze system (FFFS), and the behavioral inhibition system (BIS). Full details of the systems are provided elsewhere (Corr, 2008). Briefly, BAS relates to approach behavior, covering impulsivity and novelty seeking which is thought to underpin approach behavior and FFFS covers responses to aversive stimuli, mainly via avoidance, either defensive (fear) or escape (panic). In this revised model, BIS takes on the role of conflict resolution, and is activated whenever there is a conflict going on. This conflict may involve conflict goals between the systems (e.g., BAS– approach and FFFS – avoidance) or within a system and BIS inhibits on-going action, focuses resources and attention toward the object of the conflict, and crucially brings in the emotive response of anxiety. In terms of handedness, we have argued (Wright and Hardie, 2012) that as left-handers score themselves higher on BIS scales, but there are no hand-preference differences on the other scales, then this may hold the key to understanding behavioral differences. Supporting the role of BIS as a conflict resolution system, Smillie et al. (2007) found that measures of BIS-reactivity predicted increased response-sensitivity and response bias in goal conflict situations. In addition, BIS sensitivity is linked to a preference for familiarity, where high BIS is associated with a stronger preference for familiar images (Quilty et al., 2007). BIS is also positively associated with self-reported emotional regulation difficulties (Tull et al., 2010), suggesting that it relates to anxiety and rumination. It is important to note that anxiety serves as a mechanism to focus attention toward the conflict (Corr, 2011). BIS activation inhibits ongoing behavior, thus causing a pause in proceedings, while simultaneously directing arousal and attention toward the stimuli causing the conflict, resulting in a state of anxiety. In this context, anxiety operates as an emotional state that seeks to resolve the conflict, and is experienced in the form of worry and rumination about the source of the conflict, which increases until the point of resolution (see Corr and McNaughton, 2008). This resolution can be either an approach or avoidance. In the present case, the resolution to the conflict would be the start of the tower task, namely initiating the task, so rRST may be an explanation for the general tendency for left-handers to take longer to start a novel task.

A general difference in responsiveness between left- and right-handers is also supported by studies looking at physiological responsiveness to physical stressors. For example, Jaju et al. (2004) found in males, that when performing the cold pressor and handgrip dynamometry tests with their preferred hands, that the heart rate increase from baseline levels was significantly greater for left-handers. This suggests a possible difference in left and right-handers in their autonomic control over their cardio-vascular systems. When mental stress (i.e., cognitive load) is added in the form of a mental arithmetic task, measurement of vascular reactivity (comparing the increase from baseline to cognitive load condition) was significantly greater for left-handers including both males and females (Stoyanov et al., 2011). This suggests that when left- and right-handers are placed into stressful situations, that left-handers may show a relatively larger increase in physiological responsiveness than right-handers. However, these explanations do not fully explain all the current findings, especially the response of female left-handers on their first trial.

An alternative, or related, explanation for the state and trait anxiety findings, particularly those related to both simplicity and gender is the concept of stereotype threat or priming. Stereotype threat can be defined as an action which affects performance due to the influence of a stereotype about a specific group (Hively and El-Alayli, 2014). This is often a negative action (and can be detrimental to performance) but can also be positive (and enhance performance). The most widely cited stereotype threat literature focuses on gender stereotypes.

Research examining the relationship between anxiety and stereotype threat has found mixed results. Some studies report that self-reported anxiety is not related to performance in a stereotyped group (e.g., Schmader, 2002) while other findings show that anxiety significantly influences performance (e.g., Osborne, 2001). Bosson et al. (2004) examined different types of anxiety in relation to stereotype threat and found that those in a stereotype threat situation demonstrated more non-verbal anxiety than self-report anxiety. In addition, females’ self-reported maths anxiety was found to be influenced by gender stereotypes about maths (Goetz et al., 2013). In our study we did find that the left-handed females reported significantly higher levels of state anxiety when faced with a simple task. Although this appeared to influence their approach it was not detrimental to task performance.

The positive or negative effect of the stereotype priming on cognitive performance depends whether the participant views the testing session as challenging (Hausmann, 2014). In our study when left-handed females view the 3-disk ToH for the first time it contains two main pieces of information. The first is that it is a simple task; there are three pegs and 3-disks. The second piece of information is that it is a visuo-spatial task; participants have to move single disks through space and put them on alternative pegs until the task is solved. It is a well-known finding that males tend to outperform females on visuo-spatial tasks and are more confident in their cognitive abilities (Hausmann, 2014). Additionally females have been found to perform worse on spatial tasks when contextualized in a stereotype threat manner (i.e., informing females that they do not perform this type of task as well as males, e.g., McGlone and Aronson, 2006). Therefore we could argue that in our study, females, in general would have higher levels of state anxiety when asked to complete the ToH (which they did). However, to try to explain why *left-handed females* have the highest state anxiety levels it is proposed that the simplicity of the task could be influencing this. The 3-disk ToH is a relatively simple task, therefore the possibility of failure or not solving the simple task efficiently could influence the state anxiety levels of the left-handers. Conversely the 4-disk ToH, looks relatively more complex and thus it could be argued that the pressure to perform the task efficiently is reduced (as it is expected that it is complex and thus a minimum moves solution would be much more difficult to obtain). The level of stereotype threat could also be influenced by social factors such as people’s perceptions of performance. Therefore state levels of anxiety could be influenced by the presence of the experimenter who is observing the performance on the task.

Left-handers as a group are potentially susceptible to stereotype threat. There are many negative associations cited which could cause left-handers to become more aware of the situation and this in turn could influence both anxiety levels and task performance. Many left-handers have grown up hearing about negative connotations such as left-handedness is pathological (Satz et al., 1985), left-handers are more likely to display symptoms of depression (Denny, 2009) or left-handers have lower levels of intelligence (Hicks and Beveridge, 1978). Adding to this is popular science literature such as ‘the left-hander syndrome’ (Coren, 1993) and ‘Handedness and developmental disorder’ (Bishop, 1990). Spere et al. (2005) showed a putative link between handedness and self-consciousness, where right-handed individuals tended to have lower levels of self-consciousness, although this was only approaching significance. To date there is no literature investigating stereotype threat in left-handers but we propose that it is an interesting concept which needs to be further investigated.

### LIMITATIONS

For links to rRST, it is important to note that we did not measure BIS and future work should measure this within the context of the work, rather than rely on associations from other work. The number of participants is another limitation, as having four conditions and gender as variables there were insufficient numbers of female left-handers in the sample to allow the results to be even more finely investigated.

## Conflict of Interest Statement

The authors declare that the research was conducted in the absence of any commercial or financial relationships that could be construed as a potential conflict of interest.
